# Folate receptor-targeted nanoparticle delivery of HuR-RNAi suppresses lung cancer cell proliferation and migration

**DOI:** 10.1186/s12951-016-0201-1

**Published:** 2016-06-21

**Authors:** Ranganayaki Muralidharan, Anish Babu, Narsireddy Amreddy, Kanthesh Basalingappa, Meghna Mehta, Allshine Chen, Yan Daniel Zhao, Uday B. Kompella, Anupama Munshi, Rajagopal Ramesh

**Affiliations:** Departments of Pathology, University of Oklahoma Health Sciences Center, Oklahoma City, OK 73104 USA; Departments of Radiation Oncology, University of Oklahoma Health Sciences Center, Oklahoma City, OK 73104 USA; Departments of Epidemiology and Statistics, University of Oklahoma Health Sciences Center, Oklahoma City, OK 73104 USA; Stephenson Cancer Center, University of Oklahoma Health Sciences Center, Oklahoma City, OK 73104 USA; Graduate Program in Biomedical Sciences, University of Oklahoma Health Sciences Center, Oklahoma City, OK 73104 USA; Department of Pharmaceutical Sciences and Opthalmology, University of Colorado, Denver, CO 80045 USA; Department of Pathology, Stanton L. Young Biomedical Research Center, Suite 1403, 975 N.E., 10th Street, Oklahoma City, OK 73104 USA

**Keywords:** Folate, HuR, Lung cancer, Nanoparticle, Oncoprotein, siRNA, Targeting

## Abstract

**Background:**

Human antigen R (HuR) is an RNA binding protein that is overexpressed in many human cancers, including lung cancer, and has been shown to regulate the expression of several oncoproteins. Further, HuR overexpression in cancer cells has been associated with poor-prognosis and therapy resistance. Therefore, we hypothesized that targeted inhibition of HuR in cancer cells should suppress several HuR-regulated oncoproteins resulting in an effective anticancer efficacy. To test our hypothesis, in the present study we investigated the efficacy of folate receptor-α (FRA)-targeted DOTAP:Cholesterol lipid nanoparticles carrying HuR siRNA (HuR-FNP) against human lung cancer cells.

**Results:**

The therapeutic efficacy of HuR-FNP was tested in FRA overexpressing human H1299 lung cancer cell line and compared to normal lung fibroblast (CCD16) cells that had low to no FRA expression. Physico-chemical characterization studies showed HuR-FNP particle size was 303.3 nm in diameter and had a positive surface charge (+4.3 mV). Gel retardation and serum stability assays showed that the FNPs were efficiently protected siRNA from rapid degradation. FNP uptake was significantly higher in H1299 cells compared to CCD16 cells indicating a receptor-dose effect. The results of competitive inhibition studies in H1299 cells demonstrated that HuR-FNPs were efficiently internalized via FRA-mediated endocytosis. Biologic studies demonstrated HuR-FNP but not C-FNP (control siRNA) induced G1 phase cell-cycle arrest and apoptosis in H1299 cells resulting in significant growth inhibition. Further, HuR-FNP exhibited significantly higher cytotoxicity against H1299 cells than it did against CCD16 cells. The reduction in H1299 cell viability was correlated with a marked decrease in HuR mRNA and protein expression. Further, reduced expression of HuR-regulated oncoproteins (cyclin D1, cyclin E, and Bcl-2) and increased p27 tumor suppressor protein were observed in HuR-FNP-treated H1299 cells but not in C-FNP-treated cells. Finally, cell migration was significantly inhibited in HuR-FNP-treated H1299 cells compared to C-FNP.

**Conclusions:**

Our results demonstrate that HuR is a molecular target for lung cancer therapy and its suppression using HuR-FNP produced significant therapeutic efficacy in vitro.

## Background

Effective treatment of lung cancer continues to pose challenges resulting in a dismal overall five-year survival rate of less than 16 % [1]. Despite the availability of strong chemotherapy agents, therapeutic success is limited due to low tumor selectivity and increased non-specific cytotoxicity, the emergence of multi-drug resistance, and metastasis [2, 3]. Advances in molecular-targeted therapies have shown promise in clinical outcomes [4–6]. However, the emergence of secondary acquired mutations to the therapeutic has limited their continued use for treating lung cancer [7, 8]. Therefore, new and improved therapies are needed to overcome the existing limitations of cancer therapy.

The human antigen R (HuR), an RNA binding protein, has been implicated in the regulation of mRNAs whose protein products have key roles in cancer cell proliferation, angiogenesis, and tumor-associated inflammation [9, 10]. HuR regulation of target mRNAs is based on the interaction between the three specific domains of the HuR protein and the AU-rich elements on the untranslated region of the target mRNA [11]. Researchers have reported that HuR overexpression in various cancer types is associated with aggressive malignancy and is an indicator of poor prognosis [9, 12–14]. We and others have observed elevated expression of HuR in human-derived non-small cell lung cancer (NSCLC) cells [14–16]. Since HuR is overexpressed in cancer cells and regulates several oncoproteins, we hypothesized that small interfering RNA (siRNA)-mediated silencing of HuR will produce global knockdown of HuR-regulated oncoproteins resulting in reduced tumor cell survival.

Inhibition of HuR expression in cancer cells can be achieved with siRNA [15–18]. The high specificity and the expression-modulating capacity of siRNA make it a promising RNA interference (RNAi) tool for HuR inhibition. However, the vulnerability of siRNA therapeutics to degradation and rapid clearance in the in vivo environment warrant a safe but efficient carrier for its delivery to the target [19, 20]. Nanoparticle (NP)-based gene delivery vehicles, which include a wide range of systems made up of polymers, silica, gold particles, and other hybrid systems, are gaining more attention due to their small size, ability to overcome biological barriers, and their specific gene delivery potency [20–22]. Liposomes or lipid-based NP systems also continue to be used for gene and drug delivery and continues to hold its wide use for therapeutic applications [5, 23, 24]. Studies from our laboratory and others have previously shown that DOTAP:Cholesterol (DOTAP:Chol) lipid-based NP can efficiently deliver therapeutic nucleic acid molecules to cancer cells both in vitro and in vivo [5, 23–26]. An added advantage of DOTAP:Chol. NP is its low toxicity and high transfection efficiency, which allowed its entry into clinical testing for lung cancer therapy [27]. Hence, we hypothesized that DOTAP:Chol. NP-mediated HuR siRNA delivery in lung cancer cells will result in specific and efficient knockdown of HuR producing a therapeutic response.

To test our hypothesis, we first developed a folate-conjugated DOTAP:Chol NP system (FNP) targeted towards folate receptor-α (FRA)-overexpressing lung cancer cells. Next, we tested the therapeutic efficacy of HuR siRNA containing FNP (HuR-FNP) in vitro using human lung cancer (H1299) and normal lung fibroblast (CCD16) cells that varied in their FRA expression levels. We demonstrate HuR-FNP treatment selectively targeted and inhibited tumor cell proliferation and cell migration with minimal cytotoxicity to normal cells. Molecular studies showed HuR-FNP-mediated silencing of HuR markedly reduced the expression of HuR-regulated oncoproteins in cancer cells but not in normal cells. Our work convincingly demonstrates that HuR is a molecular target for lung cancer therapy and its suppression using HuR-FNP produced significant therapeutic efficacy in vitro.

## Results

### Physico-chemical characterization of FNP and HuR-FNP

**Fig. 1:**
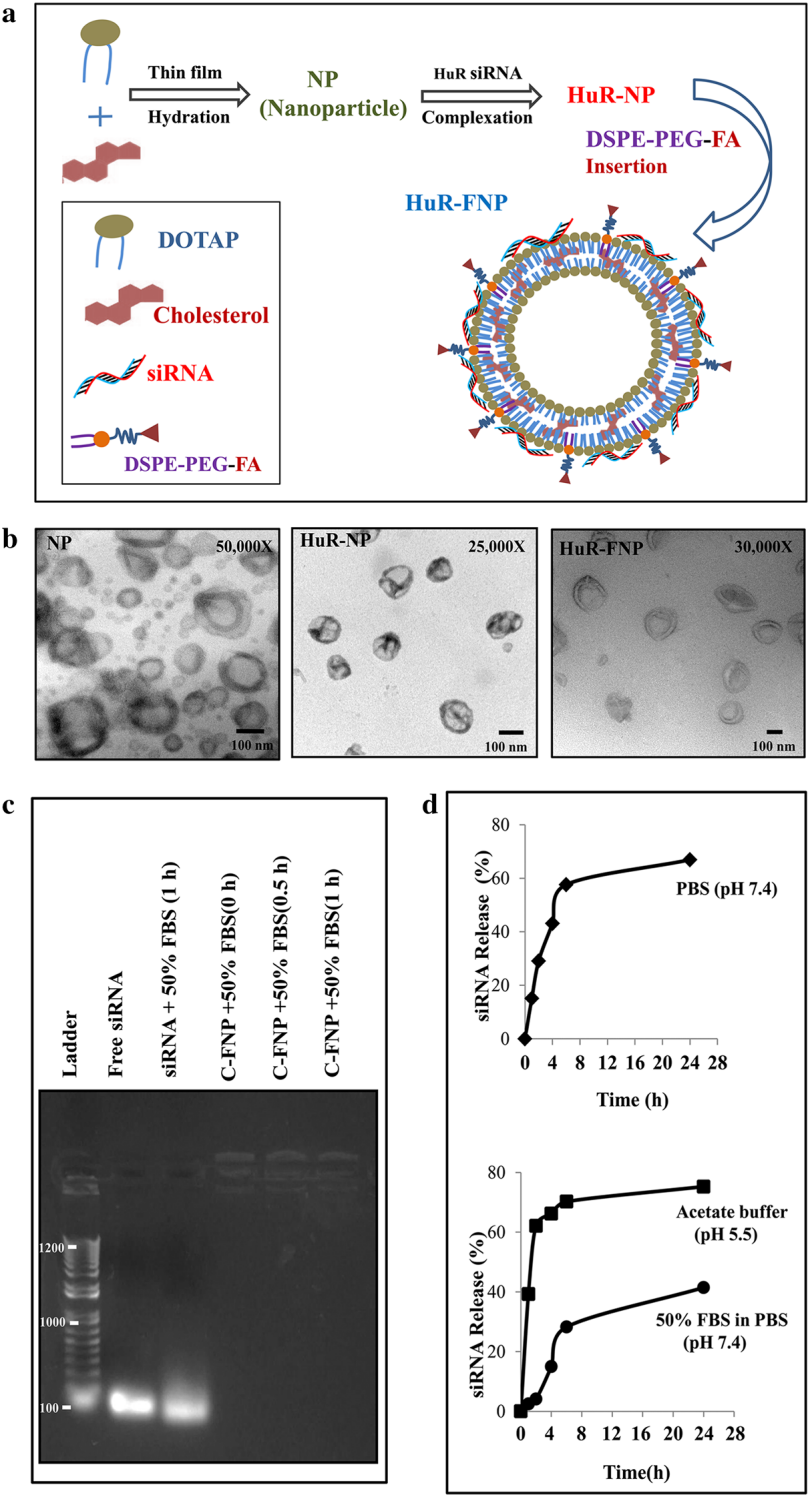
Synthesis and physico-chemical characterization of siRNA-FNP. **a** Scheme showing HuR-FNP preparation. **b** TEM image of NP, HuR-NP, and HuR-FNP. *Scale bar* denotes 100 nm. **c** Agarose gel electrophoretogram showing siRNA protection by FNP at different time (0, 0.5 and 1 h) points of incubation compared to naked siRNA exposed to serum for 1 h. Free siRNA not exposed to serum was used as internal marker. **d** siRNA release profile over time from siRNA-FNP in PBS (pH 7.4) measured by Quanti-iT Picogreen Assay (*top figure*); and from fluorescently labeled siRNA (siGLO)-FNP in acetate buffer (pH 5.5) and in 50 % FBS containing PBS (pH 7.4) [*bottom figure*]

The scheme of step-by step synthesis of HuR-FNP is depicted in Fig. 1a. FNPs prepared by the thin-film hydration method when mixed with HuR siRNA readily formed HuR-NP complexes. The HuR-FNP complex akin to HuR-NP and NP showed a near-spherical structure, as revealed by transmission electron microscopy (TEM; Fig. 1b). Table 1 shows the particle size and zeta potential values in the step-by-step synthesis of HuR-FNP. Synthesized empty NP had an average size of 158.26 nm, which increased to 205.79 nm upon HuR siRNA loading. The particle sizes obtained were within the size range of previously reported DOTAP:Chol nanoparticles that employed similar preparation procedures [28, 29]. A further increase in size (303.3 nm) was observed when DSPE-PEG_5000_-Folate was post-inserted into HuR-NP. Zeta potential of NP also varied upon loading of siRNA, from +30.89 to −2.66 mV. This remarkable change in zeta potential from strong positive to slightly negative values might be attributed to the neutralization of cationic charge by negatively charged siRNA. This finding also confirms siRNA binding to the nanoparticle. The addition of DSPE-PEG_5000_-Folate however reverted HuR-FNP complex in having a net positive charge (+4.3 mV), though the charge was still near neutral.

**Table 1:** Particle size and zeta potential of siRNA containing NPs

Components	Diameter (nm)	Zeta potential (mV)
NP	158.26	30.89
HuR-NP	205.79	−2.66
HuR-FNP (0.03 mol %)	303.3	4.3

### FNP protects siRNA from degradation

The instability of siRNA in the physiological environment due to its susceptibility to serum-nuclease catalyzed degradation, is a major limitation in RNA interference (RNAi)-based gene therapy [20]. Therefore, we studied the siRNA protection efficiency of FNP and its ability to prevent siRNA degradation in the presence of serum prior to conducting in vitro biological studies. Gel retardation assay showed that, unlike the naked siRNA that was susceptible to degradation when exposed to 50 % serum for 1 h, the siRNA contained within the FNPs was relatively intact and efficiently protected when incubated in the presence of 50 % FBS for 0.5 h to 1 h (Fig. 1c). This finding strongly suggests that the FNP is able to condense and protect the siRNA and delays the degradation by serum nucleases.

### siRNA release kinetics

To determine the release kinetics of siRNA from FNP, we conducted in vitro siRNA release profile study in PBS (pH 7.4), acetate buffer (pH 5.5) and 50 % FBS containing PBS (pH 7.4) (Fig. 1d). In general, the FNP system displayed a sustained siRNA release pattern (Fig. 1d, top and bottom figures). Around 15, 39 and 2.4 % of siRNA was released in the first hour in PBS, acetate buffer and 50 % FBS respectively. The initial high release rate over 1 h may be due to fast dissociation of loosely bound siRNA from FNP. Later at 24 h the release of siRNA reached 66, 75, and 41 % in PBS, acetate buffer and 50 % FBS media respectively. This release pattern suggested that the siRNA will be released faster under acidic conditions such as that observed in the tumor microenvironment milieu albeit some degree of degradation is likely to occur when siRNA comes in contact with serum.

### Evaluation of HuR and FRA expression levels in cell lines

**Fig. 2:**
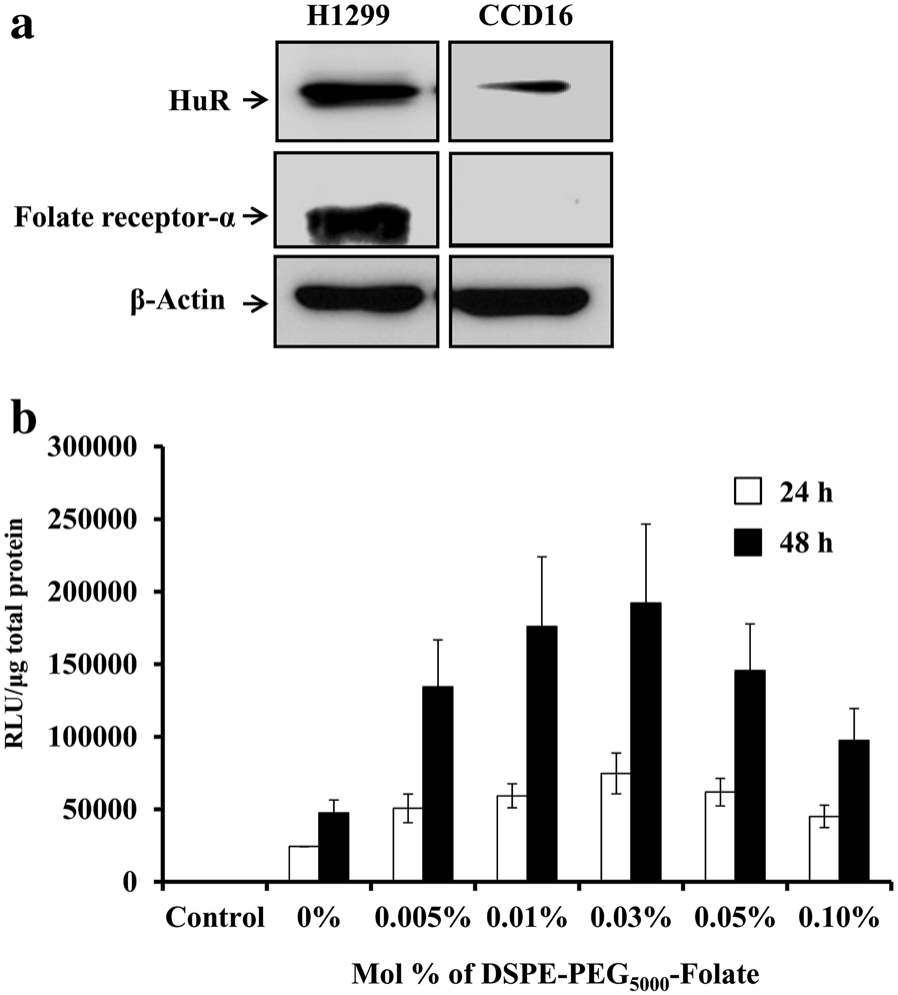
**a** Western blot showing HuR and Folate receptor-α (FRA) expression levels in H1299, and CCD16 cells. **b** Optimization of DSPE-PEG_5000_-Folate mole fraction (%) in NP was achieved by determining the luciferase activity in H1299 cells that were transfected with NP modified with varying mole fractions (%) of DSPE-PEG_5000_-Folate and carrying a luciferase-expressing plasmid. Untreated cells served as control. *Error bars* denote SD

Prior to studying the tumor-targeted delivery efficiency of HuR-FNP we determined the expression levels of FRA and HuR in H1299 and CCD16 cell lines that we have selected to use in the present study. The western blot analysis showed that the baseline HuR and FRA expression levels were high in H1299 cells compared to CCD16 cells (Fig. 2a). In fact, FRA expression in CCD16 cells was negligible.

### Mole fraction of DSPE-PEG_5000_-Folate in the NP influences transfection efficiency

The effect of various DSPE-PEG_5000_-Folate mole fractions (Mole frac. %) in the NP was evaluated for transfection efficiency in H1299 cells. NPs with varying mole % (0.005 to 0.1 %) of DSPE-PEG_5000_-Folate were formulated with luciferase-expressing plasmid DNA. Figure 2b shows that luciferase activity increased with increased DSPE-PEG_5000_-Folate concentrations, until the highest activity was reached at 0.03 mol % in H1299 cells. Luciferase activity expressed as relative light units (RLU)/μg of protein were 74,718 ± 28,129 and 192,234 ± 54,247 at 24 and 48 h respectively after treatment with NP containing 0.03 mol % of DSPE-PEG_5000_-Folate. The FNP transfection efficiency, at its optimal ligand (folate) combination, was four fold higher than that of the corresponding unmodified NP (0 %). Further increases in DSPE-PEG_5000_-Folate mole fractions (0.05 and 0.1 %) reduced luciferase activity, indicating low cellular uptake of FNP. We found that the optimal transfection efficiency occurred at 0.03 mol % of DSPE-PEG_5000_-Folate when inserted into HuR-NP. Also a further increase above 0.03 mol % of DSPE-PEG_5000_-Folate resulted in reduced transfection efficiencies in FRA expressing cells. The reduction in transfection is attributed to the increased PEG density at higher DSPE-PEG_5000_-Folate mole fractions, which likely impedes the NP interaction with the cell membrane for successful DNA delivery to occur. Our result is consistent with a previous report suggesting the influence of PEG layer thickness in electrostatic interaction between NP and cell membrane [36].

### Cell uptake of FNP corresponds to FRA expression levels

**Fig. 3:**
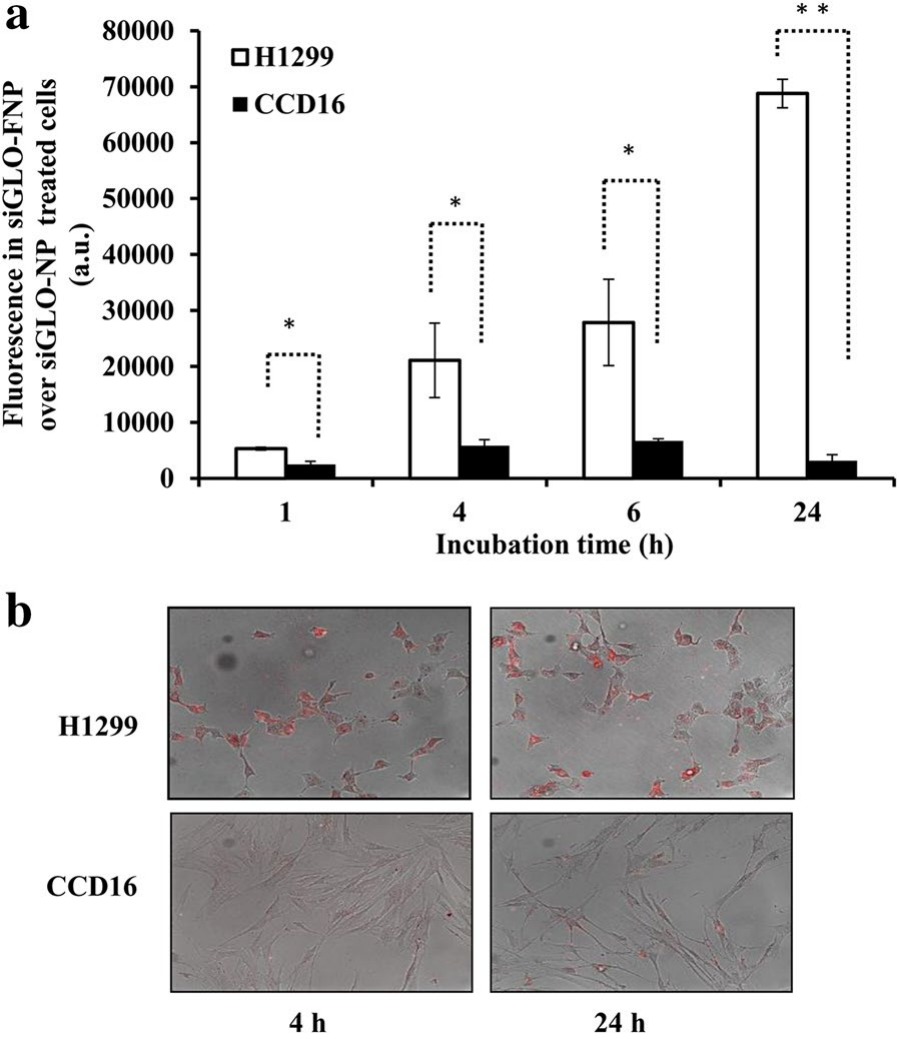
**a** SiGLO-FNP uptake over siGLO-NP over time in H1299 was significantly increased over time compared to uptake in CCD16 cells. **b** Fluorescent images of siGLO-FNP cell uptake in H1299, and CCD16 cells at 4 and 24 h post FNP treatment. (*error bars* denote SD; *p < 0.05; **p < 0.01)

To investigate the cell uptake efficiency of FNP, H1299 and CCD16 cells were transfected with FNP containing fluorescent siRNA (siGLO) and compared to NP containing siGLO (NP = no Folate ligand). The uptake of siGLO containing FNP and NP by the cells was determined over time and expressed as percent uptake of siGLO-FNP over siGLO-NP. A time-dependent increase in fluorescence was observed in siGLO-FNP-treated H1299 cells compared to CCD16 cells (*p* < 0.05; Fig. 3a) indicating enhanced siGLO-FNP uptake by tumor cells than normal cells. This observation was further supported by the live cell fluorescence images obtained using the Operetta imaging system (Fig. 3b). Enhanced siGLO fluorescence was observed in H1299 cells than in CCD16 cells both at 4 and 24 h after transfection. These results demonstrate selective uptake and delivery of siGLO-FNP occurred in FRA overexpressing H1299 cells than in low to no FRA expressing CCD16 cells.

### Enhanced uptake of FNP in H1299 cells occurs via folate receptor-mediated endocytosis

**Fig. 4:**
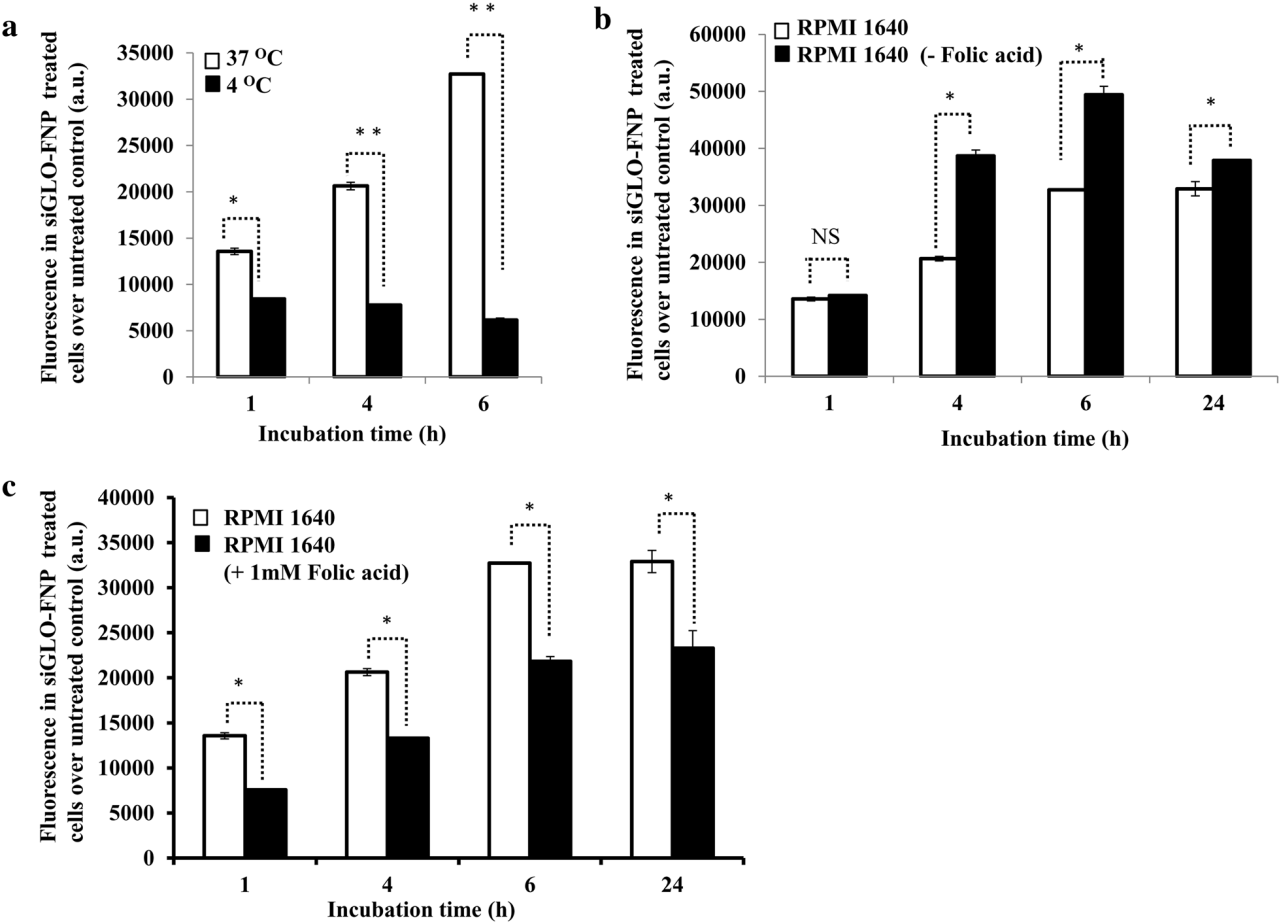
Mechanism and specificity of FNP uptake in FRA-expressing H1299 cells. **a** FNP uptake by H1299 cells, measured by fluorescence intensity of siGLO, was markedly reduced at +4 °C compared to +37 °C. **b** FNP uptake measured by fluorescence intensity of siGLO was greatly increased in the absence of folic acid in the culture medium at all time-points tested compared to FNP uptake in the medium containing trace amount of folic acid. **c** Addition of excess of exogenous folic acid (1 mM) to the culture medium reduced the FNP uptake compared to FNP uptake in the medium containing trace amount of folic acid. (*error bars* denote SD; *p < 0.05; **p < 0.01)

Studies have previously shown that folate-conjugated NPs enter the cells via receptor-mediated endocytosis [30–32]. We therefore studied the cellular internalization mechanism of FNP in folate receptor-positive H1299 cells. FNPs carrying fluorescent siRNA (siGLO-FNP) were added to H1299 cells and the uptake of siGLO-FNP was determined by incubating the cells at two different incubation temperatures (+4 and +37 °C), because temperature influences the rate of receptor-mediated endocytosis [33]. Figure 4a shows the fluorescence intensity values of siGLO expression in H1299 cells incubated with siGLO-FNP at +37 and +4 °C relative to untreated control cells. At 1 h, the fluorescence intensity of cells incubated at +37 °C was significantly higher than that of cells incubated at +4 °C (*p* < 0.05), indicating enhanced siGLO-FNP uptake. The fluorescence intensity at later time points increased from twofold to sixfold in cells incubated at +37 °C, compared with cells incubated at +4 °C (*p* < 0.01). This indicates a high rate of siGLO-FNP uptake via active receptor-mediated endocytosis at +37 °C. In contrast, the low siGLO-FNP expression observed over time at +4 °C is due to energy depletion and reduced endocytic activity. This result suggests the involvement of folate receptor-mediated endocytosis of FNP in H1299 cells.

To further test the specificity of FNP towards FRA, we carried out FNP uptake studies in three groups of H1299 cells: (1) cells incubated in regular RPMI1640 medium containing trace amounts of folic acids (indicated as RPMI1640), (2) cells incubated in RPMI1640 medium containing an excess of exogenous folic acid (+1 mM), and (3) cells incubated in RPMI1640 medium free of folic acid (–folic acid). FNP uptake by H1299 cells in folic acid-free medium was significantly enhanced (measured as fluorescence intensity of siGLO) at 4–24 h, compared with FNP uptake by cells in regular medium containing trace amounts of folic acid (Fig. 4b; *p* < 0.05). This result indicates the presence of free folic acid in the culture medium likely competes with FNP for FRA thereby affecting FNP uptake by the cells. To further test the specificity and the possibility of free folic acid competing for FRA, H1299 cells were treated with FNP in the presence of exogenous folic acid (1 mM). A marked reduction in FNP uptake and relative fluorescence intensity in H1299 cells was observed at all time points tested when excess of exogenous folic acid was present in the culture medium compared with cells in culture medium containing trace amounts of folic acid (Fig. 4c; *p* < 0.05). Our results demonstrate that the FNP specifically interacts with FRA to facilitate cell uptake and the predominant mechanism of FNP uptake occurred via receptor-mediated endocytosis.

### HuR-FNP-induced cell growth inhibition correlated with FRA expression

**Fig. 5:**
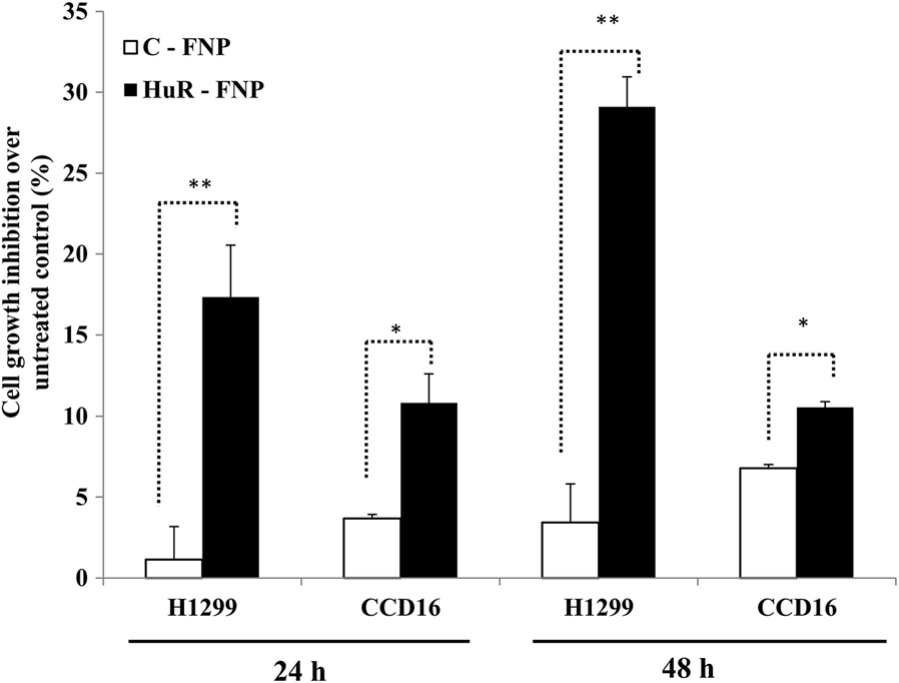
Cell growth inhibition in H1299, and CCD16 cells at 24 and 48 h after exposure to C-FNP or HuR-FNP treatment was compared with no-treatment control. (*error bars* denote SD; *p < 0.05)

siRNA-mediated HuR knockdown has been reported to induce cell apoptosis [34, 35] To assess the consistency of this observation in our studies and to evaluate whether the differential uptake of HuR-FNP complemented its cytotoxic potential in H1299, and CCD16 cells, we performed cell growth inhibition studies. Figure 5 shows the cell growth inhibition induced by C-FNP and HuR-FNP over the no-treatment controls. The cell growth inhibition induced by C-FNP and HuR-FNP in H1299 cells was 1.3 and 17.3 % respectively at 24 h and 3.4 and 29.1 % respectively at 48 h. These results demonstrate that HuR-FNP-induced inhibition was significantly larger than the inhibition observed in C-FNP-treated H1299 cells at the two time-points tested (*p* < 0.05). In the normal CCD16 cells, C-FNP and HuR-FNP treatment produced 3.6 and 10.8 % growth inhibition at 24 h and 6.7 and 10.5 % inhibition at 48 h (*p* < 0.05). Although growth inhibition was observed in HuR-FNP-treated CCD16 cells and may raise some concern, there was approximately a threefolds difference in inhibition between HuR-FNP-treated -H1299 and -CCD16 cells at 48 h indicating tumor-cell selectivity. Further, the difference in inhibition between C-FNP and HuR-FNP-treated CCD16 cells was only 3.9 % when compared to a difference of 25.7 % between the two treatment groups in H1299 cells at 48 h. These results demonstrate HuR-FNP efficiently and selectively inhibited tumor cell proliferation and the difference in the inhibitory activity observed between H1299 and CCD16 cells is attributed to differences to different levels of FRA expression.

### FRA modulates HuR-FNP-mediated tumor growth inhibition

**Fig. 6:**
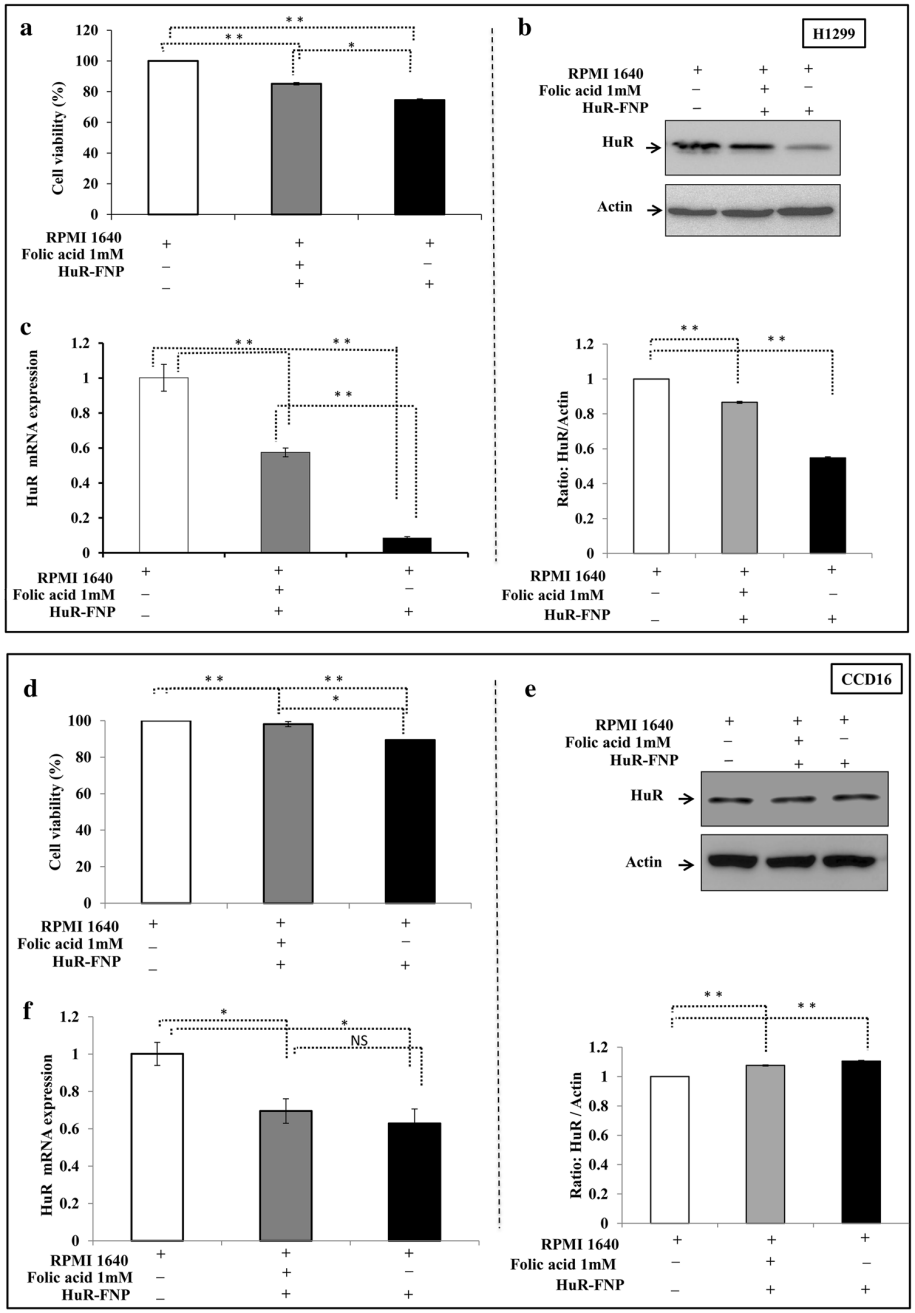
FRA modulates HuR-FNP efficacy. **a** HuR-FNP-mediated growth inhibition of H1299 cells was diminished in the presence of excess of folic acid (1 mM) in the culture medium compared to HuR-FNP inhibition in the absence of excess folic acid. HuR-FNP-mediated inhibitory activity on the expression of **b** HuR protein and **c** mRNA expression levels were also abrogated the presence of excess of folic acid (1 mM) compared to HuR-FNP treatment in the absence of excess folic acid in H1299 cells. **d** HuR-FNP-mediated growth inhibition was less in CCD16 cells that was further diminished in the presence of excess of folic acid (1 mM). **e** HuR protein and **f** mRNA expression levels were not markedly affected in CCD16 cells in the presence of excess folic acid compared to in the absence of excess folic acid. (*error bars* denote SD; *p < 0.05; **p < 0.01)

Since HuR-FNP treatment produced significant growth inhibition in H1299 cells compared to CCD16 cells, we next determined if the inhibitory activity was modulated by FRA expression and FRA-mediated HuR-FNP uptake by the cells. HuR-FNP-mediated growth inhibitory activity was markedly reduced in the presence of excess of exogenous folic acid (1 mM; 14.8 % inhibition) in the culture medium when compared to the growth inhibition in the absence of excess of folic acid (25.4 %; *p* < 0.05; Fig. 6a) in the medium. Correlating with the diminished HuR-FNP-mediated inhibitory activity in cell growth was the reduced inhibition of HuR mRNA and protein expression when excess folic acid was present in the medium (Fig. 6 b, c; *p* < 0.01). In sharp contrast, lack of folic acid in the culture medium resulted in enhanced HuR-FNP-mediated growth inhibitory activity and increased suppression of HuR protein expression in H1299 cells when compared to the HuR-FNP-treated cells grown in regular culture medium that contained trace amounts of folic acid (data not shown).

Next we determined the effect of HuR-FNP on normal CCD16 cells similar to the studies described above for H1299. HuR-FNP produced minimal cytotoxicity in CCD16 cells that was further reduced in the presence of excess of folic acid and correlated with HuR mRNA and protein expression (Fig. 6d–f). The observed results are consistent with our FNP uptake data (Figs. 3, 4) and demonstrate that HuR-FNP-mediated inhibitory activity specifically occurs via FRA and can be regulated by FRA as evidenced by the greater cytotoxicity in H1299 cells compared to CCD16 cells.

### HuR-FNP treatment suppresses HuR and HuR-regulated proteins and induces apoptosis in cancer cells

**Fig. 7:**
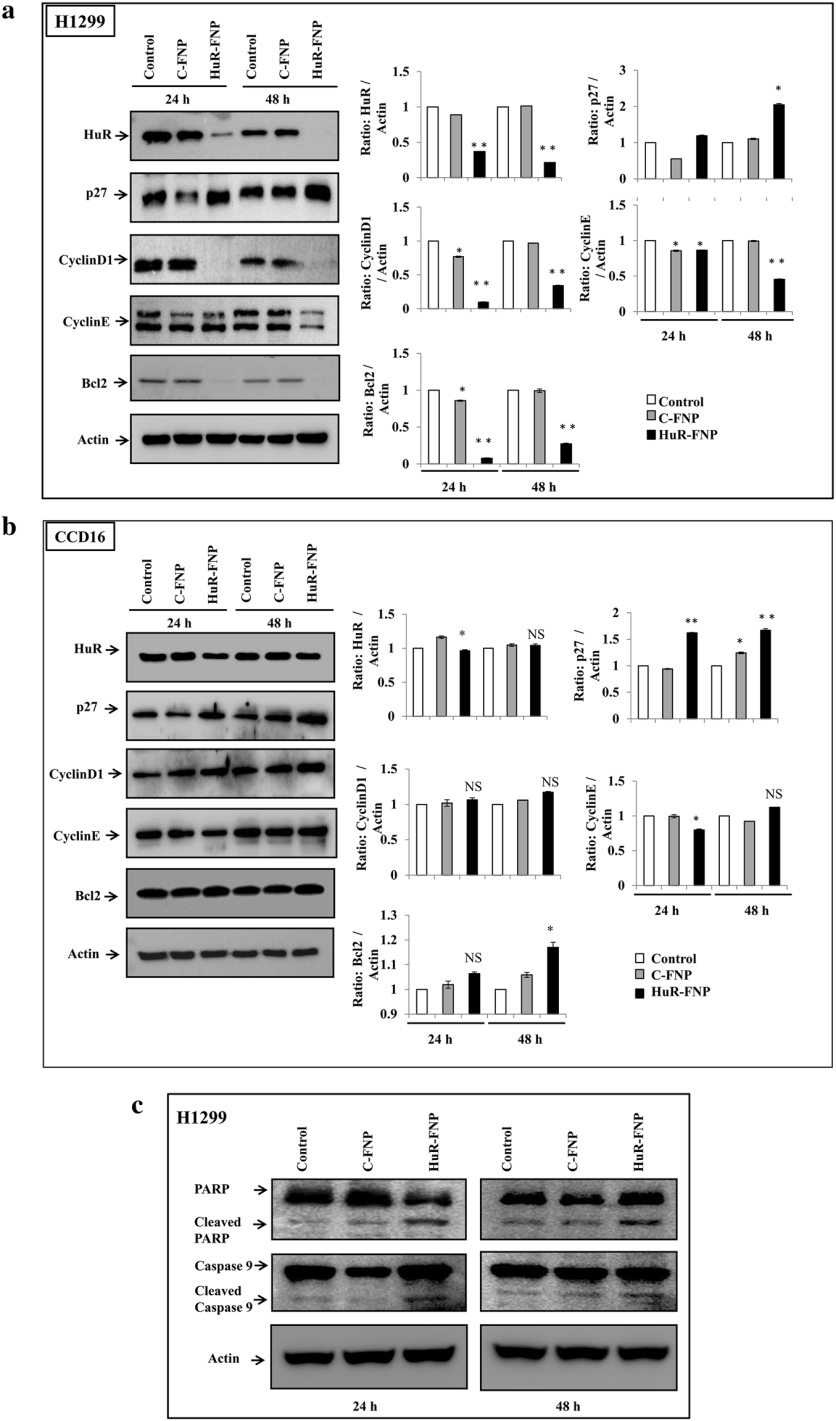
Expression of HuR, and HuR-regulated proteins in cells treated with C-FNP or HuR-FNP. Untreated cells served a control. **a** H1299, and **b** CCD16. *Bar graphs* represent semi-quantitative analysis of the protein expression detected by western blotting. Beta-actin was used as internal loading control. **c** HuR-FNP-treated H1299 cells *underwent apoptosis* as indicated by cleavage of caspase-9 and PARP at both 24 and 48 h after treatment compared to C-FNP-treated and untreated control cells. (*p < 0.05; **p < 0.01; *NS* not significant)

We next investigated the expression levels of HuR and HuR-regulated proteins (cyclin D1, cyclin E, Bcl2 and p27) in HuR-treated H1299 (Fig. 7a) and CCD16 (Fig. 7b) cell lines. In H1299 cells, HuR-FNP treatment markedly suppressed HuR that was accompanied with a concomitant decrease in cyclin D1, cyclin E and Bcl2 expression (Fig. 7a) at the two time-points tested compared to expression of these proteins in C-FNP treatment and untreated control. Expression of p27, unlike the other proteins analyzed, however was greatly increased in HuR-FNP treated cells compared to the two control groups at both 24 and 48 h (Fig. 7a). In CCD16 cells, HuR inhibition was negligible in HuR-FNP treated cells compared to C-FNP treated and untreated control cells at both 24 and 48 h. Further, no significant change in the expression of cyclin-D1 and -E, Bcl2, and p27 proteins was observed in HuR-FNP treatment compared to C-FNP treatment and untreated control (Fig. 7b). In fact, slight increase in the expression of these proteins was observed in HuR-FNP treated cells when compared to the two control groups.

Next we investigated whether silencing of HuR results in tumor cell apoptosis as previous studies have reported HuR knockdown induced cell apoptosis [34, 35]. As shown in Fig. 7c, a marked induction of apoptosis as evidenced by cleavage of caspase 9 and PARP was observed in HuR-FNP-treated H1299 cells compared to C-FNP-treated and untreated control cells. Together, our results demonstrate that HuR-FNP is capable of delivering HuR siRNA selectively to FRA expressing tumor cells and producing cell-specific knockdown of HuR and HuR-regulated target proteins resulting in tumor cell apoptosis.

### HuR-FNP induces G1 phase cell cycle arrest in lung cancer cells

**Fig. 8:**
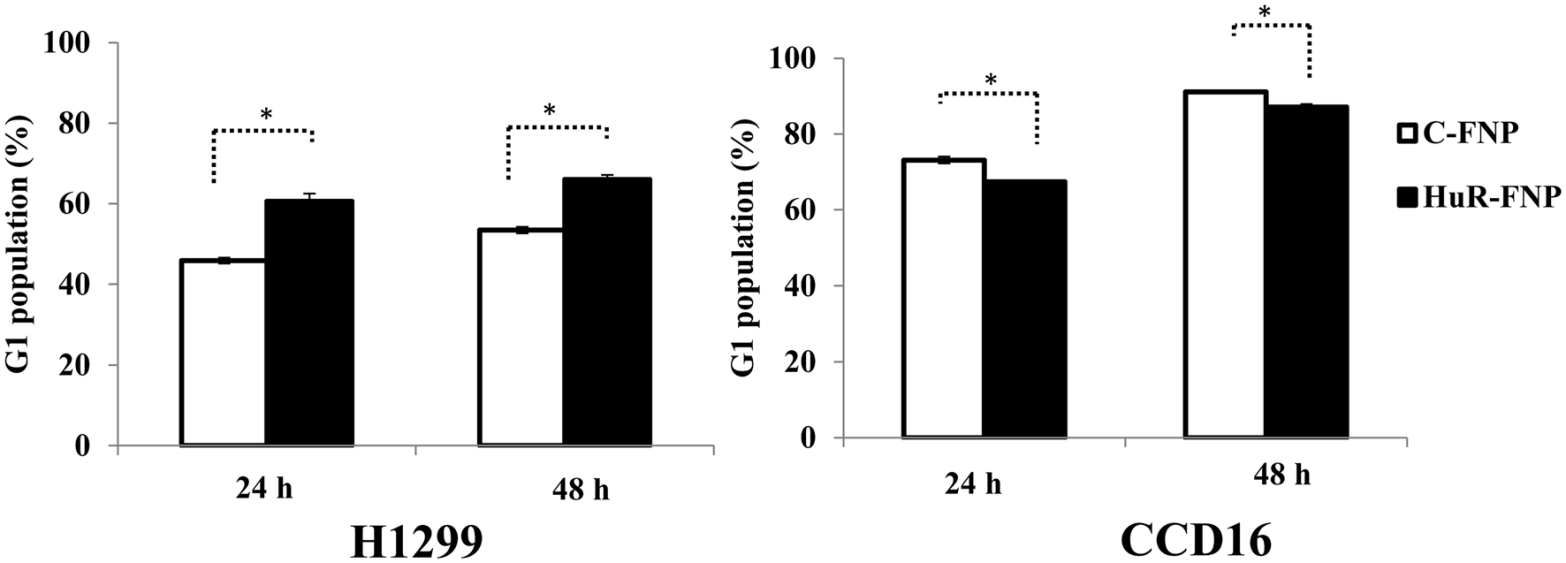
HuR-FNP induces G1 phase cell-cycle arrest and inhibits cell migration in lung cancer cells. Cell cycle analysis showed HuR-FNP induced G1 cell-cycle arrest in H1299 cells but not in CCD16 cells at both 24 and 48 h after treatment. (*p < 0.05)

In the present study HuR-FNP treatment reduced both cyclin-D1 and -E and increased p27 protein expression in H1299 cells. We therefore investigated if there were any changes in the cell-cycle phases, especially in the G1 phase, after HuR-FNP treatment. Cell cycle analysis showed HuR-FNP treatment produced a G1 phase cell cycle arrest in H1299 cells as evidenced by the marked increase in the number of cells in the G1 phase when compared to C-FNP treatment (Fig. 8; *p* < 0.05). The percent increase in the G1 phase in HuR-FNP-treated cells over C-FNP-treated cells was 15 and 12 % at 24 and 48 h. In CCD16 cells, the G1 phase was observed to decrease in HuR-FNP-treated cells compared to C-FNP-treated cells (Fig. 8; *p* < 0.05). Our results demonstrate that silencing of HuR via HuR-FNP modulates p27 and cyclin-D and -E expression resulting in G1 phase cell-cycle arrest in tumor cells but not in normal cells.

### HuR-FNP inhibits tumor cell migration

Cancer cell migration is a critical event in metastasis. Studies by us and other have previously shown that HuR regulates the expression of several oncoproteins such as actin, matrix metalloprotease (MMP)-9, urokinase-type plasminogen activator (uPA) and its receptor (uPAR), all of which are known to play an important role in cancer cell migration and invasion resulting in metastasis [16, 36, 37]. Therefore, silencing of HuR should suppress the expression of these and additional proteins and producing a net inhibitory activity on cell migration and invasion.

**Fig. 9:**
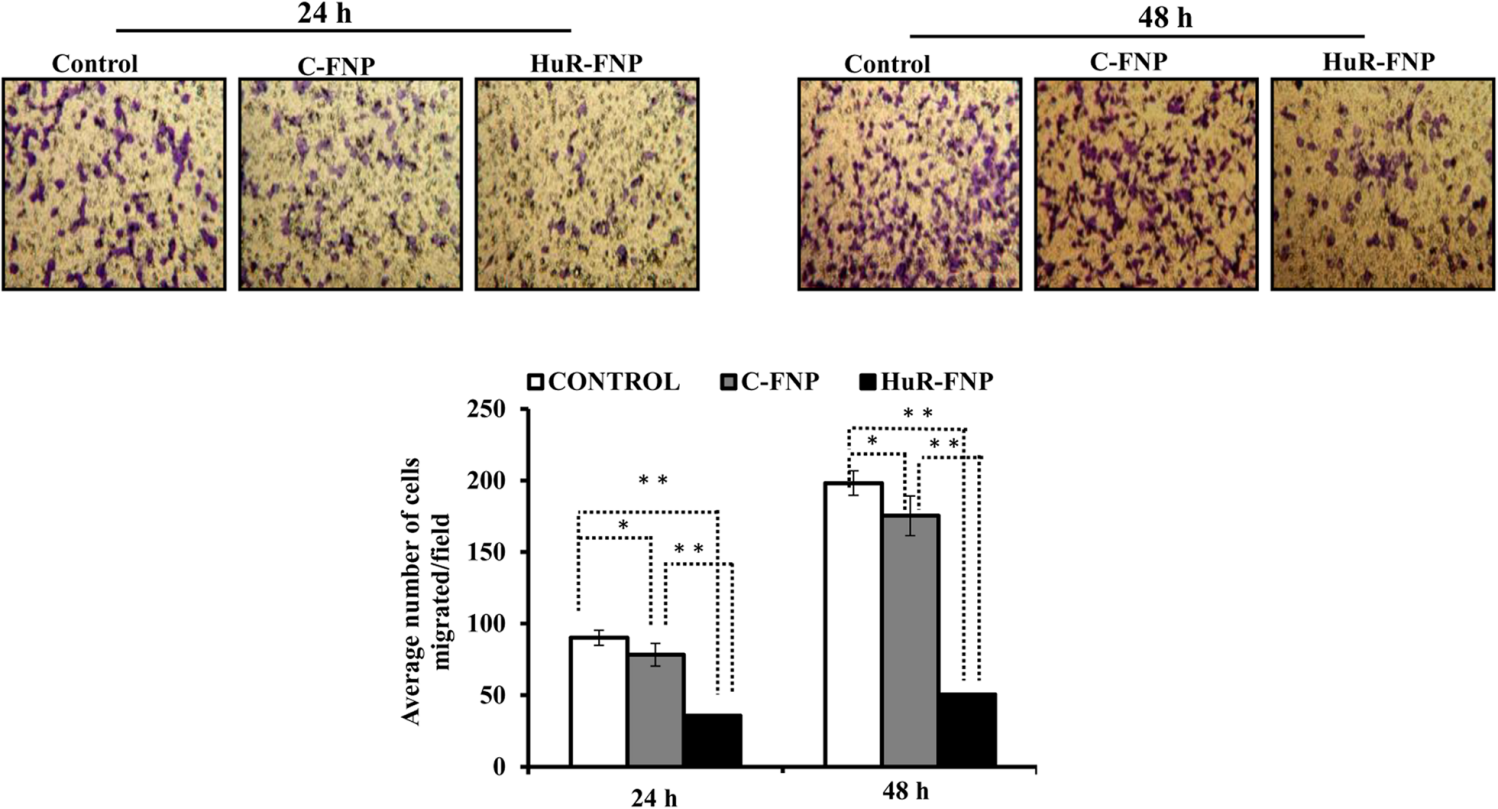
HuR-FNP treated tumor cells exhibit reduced cell migration. Cell migration studies showed that both C-FNP and HuR-FNP inhibited cell migration when compared to untreated control. However, the inhibition on cell migration exerted by HuR-FNP was greater than C-FNP at the two time-points tested. (*p < 0.05; **p < 0.01)

We therefore investigated whether HuR-FNP-mediated HuR gene silencing in H1299 cells inhibit cell migration. Cells treated with C-FNP and HuR-FNP was compared with untreated control cells for their migratory activity at 24 and 48 h. A significant inhibition of cell migration was observed in HuR-FNP-treated cells when compared to C-FNP-treatment and untreated control cells at both 24 and 48 h (*p* < 0.001; Fig. 9). The HuR-FNP-treatment reduced cell migration by 62 and 75 % at 24 and 48 h respectively when compared to untreated control. Although, inhibitory activity on cell migration was also observed in C-FNP treatment (12 and 14 % inhibition) compared to untreated control cells (*p* < 0.05), they were markedly less than that observed in HuR-FNP treatment (62 and 75 % inhibition). These results clearly demonstrate that silencing of HuR by HuR-FNP inhibited tumor cell migration.

## Discussion

In the present study we studied the efficacy of silencing HuR in lung cancer cells using FNP that is directed towards FRA overexpressing cancer cells. The rationale to use FNP was to reduce non-specific toxicity to normal cells and increase the therapeutic efficacy against tumor cells as majority of cancer therapies that are currently available are limited by cytotoxicity to normal tissues. Further, our objective to target HuR is based on prior reports from our laboratory and others demonstrating the therapeutic potential of RNAi mediated knockdown in broad-spectrum of human cancer cells [16, 38–42].

Physico-chemical characterization of the FNPs showed favorable characteristics in terms of size, shape, and charge qualifying them as a potential therapeutic nanocarrier. Further, the NPs exhibited proficient protection of the siRNA and is of prime importance as it is well known that siRNA is unstable in the physiological environment and are susceptible to serum-nuclease catalyzed degradation and thus poses a limitation in RNA interference (RNAi)-based cancer gene therapy [20]. The siRNA protection observed in the present study is likely due to the strong charge interaction between the cationic NP and anionic siRNA resulting in compaction of siRNA into the core of NP and thus protecting from unfavorable physiological components, such as the serum nucleases.

Another favorable characteristic of our FNP system was the slow and controlled release of the siRNA in physiological pH, after a high release rate observed in the initial hour of study. Slow and controlled release of siRNA is advantageous as it is likely to produce a prolonged inhibitory activity thereby reducing the frequency of treatment. The initial burst release observed at 1 h may be due to fast dissociation of loosely bound siRNA from FNP followed by slow release that could be attributed to the presence of PEG chains and folate on the NP that retards the rapid dissociation of the remained of the siRNA in the NP into the releasing media.

Biological and functional studies showed that FNP was selective and specific towards FRA expressing lung cancer cells but not towards low-no FRA expressing normal cells and that FNP uptake by the cells was receptor-mediated. Earlier studies showed that folate-conjugated NPs enter the cells via receptor-mediated endocytosis [30, 43, 44]. In this context, it was important to understand the mechanism of cellular internalization of FNP in FRA-positive H1299 cells. The rate of siGLO-FNP uptake was influenced by the incubation temperature of cells undergoing treatment. A low cellular uptake at +4 °C and a higher uptake at +37 °C of siGLO-FNP was suggestive of the involvement of receptor-mediated endocytosis, because temperature affects the energy dependent receptor-mediated endocytosis mechanism [30]. Similarly we have also proved the target specificity of FNP towards FRA overexpressing tumor cells by competitive inhibition study in the presence, absence and exogenous folic acid in the incubation media. The poor FNP uptake observed in the presence of exogenous free folic acid is attributed to competitive binding and blocking of most of the available FRA by folic acid. Altogether, our results demonstrate that the FNP specifically interacts with FRA to facilitate cell uptake and the predominant mechanism of FNP uptake occurred via receptor-mediated endocytosis.

To assess the consistency of this observation in our studies and to evaluate whether the differential uptake of HuR-FNP complemented its cytotoxic potential in H1299, and CCD16 cells, we performed cell growth inhibition studies. SiRNA-mediated HuR knockdown has been reported to induce cell apoptosis [32, 34]. Hence by knocking down HuR by HuR-FNP treatment we observed a remarkable difference in cell growth inhibition between H1299 and CCD16 cells. This result suggested that HuR-FNP efficiently and selectively inhibit tumor cell proliferation and the difference in the inhibitory activity observed between H1299 and CCD16 cells is attributed to different levels of FRA expression. Similar observation was recently reported in ovarian cancer cells where HuRsiRNA was suppressed using folate receptor targeted 3DNA nanocarriers both in vitro and in vivo [41].

Since HuR-FNP treatment produced growth inhibition in H1299 cells and not in CCD16 cells, we next investigated the expression levels of HuR and HuR-regulated proteins (cyclin D1, cyclin E, Bcl2 and p27) in the two cell lines and its effects on cell-cycle. The rationale to analyze for these proteins is based on previous reports demonstrating the presence of AU rich-elements (ARE) in the 3′ and/or 5′ untranslated regions (UTR) of the corresponding mRNAs and binding of HuR to the UTR results in mRNA regulation leading to suppression (cyclin-D1 and -E, Bcl2) or repression (p27) of these proteins [16, 34, 45, 46]. It is well known that cell cycle progression is regulated by cyclins and the associated cyclin-dependent kinases (cdks) [47, 48]. Cyclin D1 is an important activator of Cdk4 and Cdk6, which regulate cell cycle progression through the G1 phase [47]. Similarly, cyclin E is critical for cell cycle progression through the G1/S transition [49]. Another cell-cycle regulator known to be modulated by HuR is P27. P27 is a cyclin-dependent kinase (CDK) inhibitor that regulates cell-cycle by inducing cell-cycle arrest in the G1 phase [35, 46]. Further, loss of p27 expression in human cancer cells has been shown to promote cell proliferation indicating p27 also operates as a tumor suppressor [50, 51]. Studies have shown the presence of 5′ untranslated region (UTR) sequence in the p27 promoter to which HuR binds and suppress p27 protein expression [46]. However, upon inhibiting HuR, the suppressive activity of HuR is lost resulting in repression of p27 protein expression. Treatment with HuR-FNP reduced HuR protein levels in the tumor cells with concomitant reduction in cyclin-D1 and -E and increased P27 protein expression, an observation that concurred with previous reports [16, 46]. Further, changes in expression of these proteins correlated with the induction of cell cycle arrest in the G1 phase in HuR-FNP-treated tumor cells. In the normal cells no significant change in HuR or HuR-regulated proteins and cell cycle phase was observed. This observation concurs with previous reports demonstrating HuR inhibition in cancer cells produces G0/G1 phase arrest [16, 32, 45].

The ability of HuR-FNP to reduce Bcl2, an anti-apoptotic protein that has been shown to play a key role in cell survival and contribute to resistance against cytotoxics [34, 50], provides new opportunity for testing HuR-FNP efficacy in Bcl-2 overexpressing drug-resistant cancer cells. Further, combinatorial siRNA therapies against HuR and Bcl2 can be envisioned that should produce an enhanced anticancer activity. While these concepts are of interest and of relevance to cancer treatment, they are beyond the scope of the present study.

Finally, we have shown HuR suppression by HuR-FNP inhibited tumor cell migration, a phenomenon that was consistent with previous reports [16, 52, 53]. The mechanism by which HuR regulates tumor cell migration and invasion is documented to involve several oncoproteins such as actin, matrix metalloprotease (MMP)-9, urokinase-type plasminogen activator (uPA) and its receptor (uPAR), and the CXCR-4 chemokine receptor all of which are known to play an important role in cancer cell migration and invasion resulting in metastasis [16, 53, 54].

Together, our results convincingly demonstrate that HuR-FNP is capable of delivering HuR siRNA selectively to FRA expressing tumor cells and producing cell-specific knockdown of HuR and HuR-regulated target proteins producing minimal cytotoxicity to normal cells. Our exciting in vitro study results provide an impetus for testing HuR-FNP in vivo as monotherapy and as combinatorial therapy using conventional chemotherapeutics and molecularly targeted therapeutics for lung cancer.

## Conclusion

In the present study we have developed and tested the feasibility of a tumor-targeted nanoparticle siRNA delivery system for selectively targeting tumor cells and silence HuR, and establish HuR as a molecular target for lung cancer therapy. We demonstrated HuR-FNP is preferentially taken up by FRA overexpressing lung cancer cells and produce tumor cell cytotoxicity by inhibiting HuR with minimal cytotoxicity to normal cells. The antitumor activity of HuR-FNP is produced by a combination of cell growth inhibition, cell cycle arrest, suppression of oncoproteins, and impeded cell migration. While our study results are exciting, it is to be noted that additional preclinical studies are warranted prior to clinical testing. Our study results also provide an avenue for conducting combinatorial therapy studies against drug-resistant cancer cells. Finally, HuR-FNP-based cancer therapy can be applied in treating a broad-spectrum of human cancers that are known to overexpress HuR.

## Methods

### Materials

1,2-dioleoyl-3-trimethylammonium-propane chloride (DOTAP), cholesterol, and 1,2-distearoyl-*sn*-glycero-3-phosphoethanolamine-N-[folate(polyethylene glycol)-5000] (DSPE-PEG_5000_-Folate) were purchased from Avanti Polar Lipids (Alabaster, AL, USA). Folic acid was purchased from Sigma-Aldrich Chemicals (St. Louis, MO, USA). RPMI-1640 medium, Hams-F12 medium, folic acid-free RPMI medium, and fetal bovine serum (FBS) was purchased from GIBCO BRL Life Technologies (New York, NY, USA). Luciferase assay reagent and lysis buffers were obtained from Promega (Madison, WI). Control siRNA (5′ UAA GGC UAU GAA GAG AUA C 3′), and HuR-siRNA (5′ UCA AAG ACG CCA ACU UGU A 3′) were purchased from Dharmacon (Lafayatte, CO). Luciferase (luc) expression plasmid vector was obtained from Clonetech (Mountain View, CA, USA).

### Cell lines

Human non-small-cell lung carcinoma (NSCLC) cell line (H1299) and human normal lung fibroblast cell line (CCD16) were obtained from American Type Culture Collection (ATCC, Rockville, MD, USA). Cells were maintained in appropriate culture medium as previously described [16]. The cell lines were authenticated to be of human origin by single tandem repeat assay (STR; IDEXX Bioresearch, Columbia, MO, USA).

### Synthesis of cationic lipid nanoparticles

Cationic lipid nanoparticles (NPs) were synthesized using the thin-film hydration method as previously described [55]. Briefly, a 20 mM equivalent of DOTAP and Chol was mixed and dissolved in chloroform. A thin DOTAP:Chol film was created in a round-bottom flask with the help of a Rotavapor® under ambient conditions, and was vacuum-dried. Dextrose (5 %) in sterile water (D5W) was used as hydration solution to create liposomes from the dried lipid film. The liposomes were then sequentially extruded through an Avanti® Mini-Extruder (filters with 1–0.1 μM pores) to produce liposomes with reduced particle size and low polydispersity. For preparation of DNA- or siRNA-containing NPs, diluted DOTAP:Chol stock solution and DNA or siRNA solution in D5 W was mixed in equal volumes to give a final concentration of 4 mM DOTAP:Chol containing 1 µg DNA or 100 nM siRNA equivalent per well of a six-well plate.

### Preparation of folate-conjugated NP

DSPE-PEG_5000_-Folate was inserted into preformed DNA- or siRNA-containing NPs using the post-insertion technique [16]. The post-insertion mechanism involves the insertion of acyl chains of DSPE phospholipid into the DOTAP:Chol lipid bilayer as a result of hydrophobic–hydrophobic interaction and has shown to be a successful method of lipid NP modification [43]. While addition of PEG chains (via DSPE-PEG) has successfully been shown to impart long circulating properties to NP [44], folate contributes to their targeting ability.

Briefly, a stock solution of DSPE-PEG_5000_-Folate (17 µM) was prepared by thin film hydration. In the next step, various concentrations of DSPE-PEG_5000_-Folate (0.005, 0.01, 0.03, 0.05, or 0.1 mol fraction % of DOTAP:Chol) were mixed with DNA- or siRNA-containing NP by vigorous pipetting. Pipetting was followed by incubation at room temperature (RT) for 60 min to form FNPs. The FNPs were then dialyzed against distilled water overnight at 4 °C. After dialysis, the FNP suspension was stored in sterile 1-ml screw-cap tubes at 4 °C. The tubes were labelled as HuR-FNP and C-FNP for FNP carrying HuR siRNA and control siRNA, respectively. Unmodified NPs were synthesized without the addition of DSPE-PEG_5000_-Folate.

### Nanoparticle characterization

#### Size, shape, and zeta potential

Particle size distribution and zeta potential of the NP formulations were determined using the Brookhaven ZetaPALS instrument. The shape and size of the NPs stained by uranyl acetate (2 % for 5 min) were analyzed using a Hitachi H-7600 transmission electron microscope (TEM) at the Oklahoma Medical Research Foundation (OMRF) core facility.

#### siRNA protection assay

An electrophoretic mobility assay of siRNA-loaded NP formulations was conducted to determine whether FNP formulations protect siRNA in the presence of serum [16]. Briefly, siRNA-containing FNPs were incubated with 50 % FBS at 37 °C for 0, 30, and 60 min, respectively. Then, an aliquot of each sample was collected and was subjected to gel electrophoresis in 1.2 % agarose gel at 100 V for 30 min in Tris-acetate-EDTA buffer (TAE; pH 8.0). The efficiency of siRNA protection by FNP was assessed by examining the gel for retardation of encapsulated siRNA in the wells, and comparing this with migrating free siRNA control. The gel was stained with ethidium bromide (Sigma Chemicals). Images were captured using a gel documentation system (Syngene, Frederick, MD, USA).

#### siRNA release profile

100 μl of siRNA (100 nM) loaded FNP was suspended in phosphate-buffered saline (PBS; pH 7.4) and incubated at 37 °C with gentle shaking at 120 rpm. At predetermined time points, the FNP suspension in PBS was centrifuged for 10 min at 12,000*g*. The supernatant was removed and the pellet was resuspended in fresh PBS. The collected supernatant was reacted with Quant-iT Picogreen reagent® (Thermo Fisher Scientific, USA) per the manufacturers’ protocol. The reaction product was subjected to fluorescence measurement at 485 nm excitation and 535 nm emission wavelengths using an EnVision® multilabel reader (Perkin Elmer, Santa Clara, CA, USA). The amount of siRNA in the releasing media was quantified and represented as the percentage of siRNA encapsulated in FNP.

Further, to show the release profile of siRNA in low pH conditions and also in physiological pH in the presence of serum, we carried out separate in vitro release experiments using fluorescently labelled siRNA (siGLO) loaded FNP. Briefly, siGLO-FNP (200 μl) samples were suspended in acetate buffer (pH 5.5) and 50 % fetal bovine serum (FBS) containing PBS (pH 7.4) in separate vials and incubated at 37 °C with gentle shaking at 120 rpm. At predetermined time intervals the siGLO-FNP samples were centrifuged (12000×*g*; 15 min) and the supernatants were withdrawn and replaced with respective (fresh) buffers. The supernatants were analyzed using Envision Multilabel reader at 557 nm excitation and 570 nm emission wavelengths for siGLO. The amount of siRNA release was quantified and represented as percentage of siRNA released from FNP.

### Folate optimization

To choose the NP formulation with the optimal targeting and transfection efficiency, luciferase plasmid DNA carrying NPs decorated with various DSPE-PEG_5000_-Folate mol fractions (0.005, 0.01, 0.03, 0.05 %, or 0.1 mol fraction %) were prepared and tested in vitro. Briefly, H1299 cells (1 × 10^5^ cells/well) were seeded in six-well plates and were transfected with FNPs carrying luciferase reporter plasmid DNA (1 μg/well) in serum-free medium. After 6 h of transfection, the medium was replaced with RPMI-1640 containing 2 % FBS and incubation was continued at 37 °C, 5 % CO_2_. The cells were harvested at 24 and 48 h after transfection. Luciferase expression was determined using a luciferase assay kit (Promega, Madison, WI, USA) as previously described [16, 37]. Cells grown without treatment and cells treated with DSPE-PEG_5000_-Folate-free (unmodified or 0 % folate) NPs served as controls.

### Cellular uptake of FNP

Lung cancer (H1299) and normal lung fibroblast (CCD16) cells that have differential expression of folate receptor were chosen for studying the cellular uptake of FNP. Briefly, 1 × 10^5^ cells/well was seeded in six-well plates. The cells were transfected with either NP or FNP that were loaded with fluorescently labelled siRNA (siGLO, Dharmacon; 100 nM equivalent) in serum-free medium. The cells were harvested at various time (1, 4, 6, and 24 h) points after transfection. The cellular uptake of FNP was determined quantitatively using an Envision multilabel plate reader and qualitatively using the Operetta imaging system (Perkin Elmer).

To determine whether the uptake of FNP is facilitated by receptor-mediated endocytosis, H1299 cells (1 × 10^5^ cells/well) were transfected with FNP containing fluorescently labelled siRNA (siGLO, 100 nM) and incubated for various time (1, 4, and 6 h) points at either 37 or 4 °C. After the incubation period, the cells were washed with PBS, were harvested, and were analyzed with an Envision multiple plate reader.

To determine the role of folate receptor in FNP internalization, cell uptake studies were conducted in the presence and absence of free folic acid in culture medium and with or without 1 mM exogenous folic acid. The experimental setup used H1299 cells (1 × 10^5^ cells/well) grown for 24 h in RPMI medium, with or without folic acid. The next day, cells were transfected with FNP containing fluorescently labelled siRNA (siGLO, 100 nM). At various time (1, 4, 6, and 24 h) points after transfection, the cells were harvested and were analyzed quantitatively using an Envision multilabel plate reader.

### Cell viability assay

The efficiency of HuR-FNP in lung cancer cell growth inhibition was determined using the standard Trypan blue exclusion assay method as previously described [16, 55, 56]. Briefly, cells (H1299, CCD16; 1 × 10^5^ cells/well) seeded in six-well plates were treated with FNP containing control siRNA (C-FNP) or HuR-FNP (100 nM siRNA) in serum-free medium. After 6 h of incubation, the medium was replaced with 10 % FBS-containing medium. Cells that did not receive any treatment served as control. At 24 and 48 h after treatment, the cells were harvested and the number of viable cells was counted. The results were expressed as percentage inhibition over untreated control cells. The experiments were repeated three times for reproducibility and were analyzed statistically.

### Quantitative (Q) RT-PCR assay

Total RNA from FNP treated cells was isolated using Trizol reagent (Life Technologies, Grand Island, NY, USA) and the RNA quality was determined using Denovix DS11 spectrophotometer as previously described [37]. Complimentary DNA (cDNA) was synthesized from RNA (2 µg/sample) using a Quant script cDNA synthesis kit (Bio-Rad, Richmond, CA, USA). Then, 3 µl of cDNA was amplified by real-time (RT)-PCR (Bio-Rad CFX96™ TouchReal-Time PCR Detection System) using the premix iQ SYBR green QRT-PCR kit (Bio-Rad) and human HuR-specific oligonucleotide primers (Forward- 5′ ATGAAGACCACATGGCCGAAGACT 3′- Sense; Reverse-5′ TGTGGTCATGAGTCCTTCCACGAT 3′-Antisense). Thermal cycling was programmed as follows: 50 °C for 2 min, 95 °C for 3 min, then 40 cycles of 95 °C for 10 s alternating with 60 °C for 10 s and 72 °C for 30 s. The cycle threshold (Ct) value assessed by RT-PCR was noted for the transcripts and was normalized with human GAPDH (Forward-5′AGCCTCAAGATCATCAGCAATGCC 3′ and Reverse- 5′ TGTGGTCATGAGTCCTTCCACGAT 3′). The changes in mRNA expression levels were expressed as fold change relative to control ± standard deviation (SD). Each sample was run in triplicate. The experiments were repeated at least twice for reproducibility and statistical calculation.

### Cell cycle analysis

Cells (H1299, CCD16) were transfected using C-FNP and HuR-FNP (100 nM siRNA) as described in the cell viability assay above. At 24, and 48 h after treatment, the cells were collected, processed and were subjected to flow cytometric analysis as previously described [26, 37].

### Western blotting

Total protein extracted from cells that underwent various treatments was subjected to western blot analysis as previously described [15, 37, 56]. Briefly, protein samples were separated in 10 % SDS polyacrylamide gel and were transferred to a polyvinylidene fluoride (PVDF) membrane (Immobiloin®, Millipore, MA, USA). After the transfer, the membranes were blocked in 5 % milk containing buffer (1X Tris buffered saline with Tween 20^®^ [TBST]) for 30 min. In the following step, membranes were incubated with primary antibodies against human HuR, p27, cyclin D1, cyclin E, Bcl-2 (Santa Cruz Biotechnology, Dallas TX, USA), Folic acid receptor-alpha (GeneTex Inc., Irvine, CA, USA), caspase-9 and PARP (Cell Signaling Technology Inc., Beverly, MA, USA), and beta-actin (Sigma-Aldrich, St. Louis, MO, USA), respectively, in 5 % milk containing TBST. Protein bands were detected using appropriate horseradish peroxidase-(HRP)-tagged secondary antibodies (Santa Cruz Biotechnology) and an enhanced chemiluminescence kit (Thermo Scientific, MA, USA). Protein expression levels were detected using a chemiluminescence imaging system (Syngene, Frederick, MD, USA) and quantified using Gene tools (Syngene) software [37].

### Cell migration assay

Cell migration assay was carried out as previously described [16, 57]. Briefly, H1299 cells (5 × 10^4^) were seeded in the upper chamber of the transwell (8 µm; BD Biosciences, Bedford, MA, USA), and were placed in individual wells (lower chamber) of six-well plates filled with 1 ml of serum-free RPMI-1640 medium. Cells were allowed to adhere for 24 h in the upper chamber, and were then transfected with C-FNP and HuR-FNP (100 nM siRNA dose) in serum-free medium. After 6 h of transfection, the medium in the upper and lower chambers was replaced with 2 and 20 % serum-containing medium, respectively. The incubation continued until the inserts were removed and were processed at 24 and 48 h, as previously described [16, 57]. The results were expressed as an average number of migrated cells per microscopic field. The experiments were performed three times for reproducibility and statistical calculation.

### Competitive inhibition studies

H1299 cells (1 × 10^5^ cells/well) were grown in six-well plates and were incubated for 1 h in RPMI-1640 medium containing excess of exogenous folic acid (1 mM). The cells were then transfected with HuR-FNP and were harvested after 24 h incubation. The harvested cells were used for cell viability measurements, western blot analysis, and qRT-PCR assays.

In separate set of experiments, H1299 cells (1 × 10^5^/well) were grown in RPMI medium containing 10 % FBS, with and without folic acid. After 24 h of incubation at 37 °C, the cells were treated with HuR-FNP in serum-free RPMI medium, with and without folic acid. After 6 h incubation for transfection, medium was replaced with 2 % serum containing RPMI medium, either with or without folic acid. The cells were harvested at 24 h after HuR-FNP treatment and were used in cell viability, western blotting, and qRT-PCR assays.

### Statistical analysis

Unless otherwise stated, all data are shown as mean ± standard deviation (SD). Univariate statistical significance was determined by one-way analysis of variance (ANOVA) with Tukey’s adjustment for pairwise comparisons. Differences between groups were obtained using a linear mixed effects model with Tukey’s adjustment. A *P* value of less than 0.05 was considered statistically significant. SAS 9.2 software was used for the statistical analyses.

## Abbreviations

HuR: human antigen R
NSCLC: non-small cell lung cancer
siRNA: small interfering RNA
RNAi: RNA interference
NP: nanoparticle
DOTAP: 1,2-dioleoyl-3-trimethylammonium-propane chloride
DOTAP:Chol: DOTAP:Cholesterol
DSPE-PEG_5000_-Folate: 1,2-distearoyl-*sn*-glycero-3-phosphoethanolamine-N-[folate(polyethylene glycol)-5000]
FRA: folate receptor-α
FNP: folate-conjugated DOTAP:Chol nanoparticle
C-FNP: FNP containing control siRNA
HuR-FNP: HuR siRNA containing FNP
cDNA: complimentary DNA
TEM: transmission electron microscopy
UTR: untranslated regions
CDK: cyclin-dependent kinase
(MMP)-9: matrix metalloprotease
uPA: urokinase-type plasminogen activator
TAE: tris–acetate-EDTA buffer
PBS: phosphate-buffered saline
